# Transcript Profiling of *Paoenia ostii* during Artificial Chilling Induced Dormancy Release Identifies Activation of GA Pathway and Carbohydrate Metabolism

**DOI:** 10.1371/journal.pone.0055297

**Published:** 2013-02-06

**Authors:** Shupeng Gai, Yuxi Zhang, Chunying Liu, Yang Zhang, Guosheng Zheng

**Affiliations:** College of Life Sciences, Qingdao Agricultural University, Key Lab of Plant Biotechnology in Universities of Shandong Province, Qingdao, China; Cankiri Karatekin University, Turkey

## Abstract

Endo-dormant flower buds must pass through a period of chilling to reinitiate growth and subsequent flowering, which is a major obstacle to the forcing culture of tree peony in winter. Customized cDNA microarray (8×15 K element) was used to investigate gene expression profiling in tree peony ‘Feng Dan Bai’ buds during 24 d chilling treatment at 0–4°C. According to the morphological changes after the whole plants were transferred to green house, endo-dormancy was released after 18 d chilling treatment, and prolonged chilling treatment increased bud break rate. Pearson correlation hierarchical clustering of sample groups was highly consistent with the dormancy transitions revealed by morphological changes. Totally 3,174 significantly differentially-expressed genes (P<0.05) were observed through endo-dormancy release process, of which the number of up-regulated (1,611) and that of down-regulated (1,563) was almost the same. Functional annotation of differentially-expressed genes revealed that cellular process, metabolic process, response to stimulus, regulation of biological process and development process were well-represented. Hierarchical clustering indicated that activation of genes involved in carbohydrate metabolism (Glycolysis, Citrate cycle and Pentose phosphate pathway), energy metabolism and cell growth. Based on the results of GO analysis, totally 51 probes presented in the microarray were associated with GA response and GA signaling pathway, and 22 of them were differently expressed. The expression profiles also revealed that the genes of GA biosynthesis, signaling and response involved in endo-dormancy release. We hypothesized that activation of GA pathway played a central role in the regulation of dormancy release in tree peony.

## Introduction

Tree peony (*Paeonia suffruticosa* Andrews) is one of the earliest and most well-known horticultural and medicinal plants in the world. Forcing culture is one of the main production parts in tree peony industry, especially for Spring Festival market in China. The dormancy is a major obstacle to the forcing culture of tree peony in winter. The flower buds of tree peony which finished differentiation in July or August must go through a period of bud dormancy before bud sprouting in spring with natural culture, or winter by forcing culture [1].

The dormancy of tree peony belongs to endo-dormancy according to Lang and Martin [2], similar to some temperate fruit plants like grape, apple, peach, kiwifruit and so on. The major method for budbreak in many perennial plants is to supply sufficient chilling exposure time during winter, and then induce growth in the following spring with appropriate warmer environment. The chilling requirement fulfillment is also important in tree peony production [1]. In recent years, warm winter appears more frequently due to global warming tendency, which results in insufficient chilling in almost all commercial cultivars. Insufficient chilling can subsequently lead to non-uniform bud break or delay bud break and flower blossom, which greatly influences the ornamental and commercial value of the flower. Therefore, more effective cultivation strategies and genetic control of dormancy release are now required, and the first step is to well understand the mechanism of dormancy induction and release.

Induction and release of dormancy were controlled through cooperation of large number of genes in woody plants [3]. Many research works about the physiological and molecular aspects of bud dormancy in grapes, kiwifruit, apple, peach and other woody plants have been published [4]–[11]. Characterization of complex network of biochemical and cellular processes for the regulation and execution of bud -dormancy release in tree peony has not yet been reported. Differences might exist in the dormancy release mechanism between tree peony and the fruit plants. For example, fruit crop was sensitive to HC (hydrogen cyanamide) which could promote the breaking of dormancy [12], but insensitive to exogenous gibberellins [13]. On the other hand, tree peony seems to be insensitive to HC according to our recent work (unpublished), while GA_3_ (gibberellin acid 3) can effectively stimulate the breaking of dormancy, which is also an important means practice applied in forcing culture. In grape, Ophir et al suggested that HC and heat shock (HS) induced dormancy release involving in tricarboxylic acid cycle (TCA cycle), ATP synthesis, oxidative phosphorylation and oxidative stress [7]. Regulations of GA biosynthesis and signaling pathway were poorly described in related studies during dormancy release after chilling and dormancy-breaking compound treatments. Hence, better understanding of physiological processes involved in dormancy regulation, especially the regulations of GA biosynthesis and signaling pathway would be benefit to understand the mechanism of dormancy release.

In recent years, studies on tree peony had been focused on physiological and horticultural aspects of dormancy, such as nutrient substance, membrane lipid peroxidation and the endogenous hormones dynamic changes during the chilling requirement fulfillment [14]–[16]. Huang et al screened 31 unigenes associated with dormancy release in tree peony by SSH (suppression subtractive hybridization) [17], [18], but the information was still limited to understand the complex biochemical network responsible for the regulation and execution of the dormancy process. More recently, we performed transcriptome sequencing in tree peony during chilling requirement fulfillment on the Roche 454 GS FLX platform, and obtained over 15,000 high quality unigenes [19]. This dataset made it feasible to investigate the levels of genome-wide gene expression in dormancy release by microarray technology and can provide more information to understand the genetic and physiological changes during the dormancy release process in tree peony.

## Materials and Methods

### Plant Materials

Four-year-old tree peony plants (*Paoenia ostii* ‘Feng Dan Bai’) were potted and moved to refrigerating room with temperature at 0–4°C from 5 November to 30 December 2009 in Qingdao, Shandong, China. Mixed buds, three buds for each individual, were collected after 0 d, 6 d, 12 d, 15 d, 18 d and 24 d chilling requirement fulfilling during the period of bud dormancy release. Three replicates (3 plants/replicate) were harvested and immediately frozen in liquid nitrogen and stored at −80°C until use.

### Measurement of Bud Dormancy Status

Tree peony plants after 0 d, 6 d, 12 d, 15 d, 18 d and 24 d chilling treatment were then transported to green house (18–22°C, 8-h-light/16-h-dark cycle). The morphological changes of mixed buds were observed to define the stages of dormancy [17], [18]. Bud break was defined as tissue visible expanding beneath bud scales. Flower buds from ten individuals were monitored for each chilling treatment, and the percentage of bud break was calculated for three replications. Analysis of variance (ANOVA) was used to determine significance of bud break and flowering rate difference (CoHort, Monterey, CA, USA).

### RNA Extraction

Total RNA of three biological replicates was extracted using TRIZOL Reagent (Carlsbad, CA, US) following the manufacturer’s instructions and RNA integrity was checked for RIN (RNA Integrity Number) by an Agilent Bioanalyzer 2100 (Agilent technologies, Santa Clara, CA, US). Qualified total RNA was further purified by RNeasy mini kit (QIAGEN, GmBH, Germany).

### Microarray Processing and Analysis

The *P. ostii* DNA microarrays were customized using Agilent eArray 5.0 program according to the manufacturer's recommendations (https://earray.chem.agilent.com/earray/). Each customized microarray (8×15 K) contained spots of 14,951 gene-specific *60-mer* oligonucleotides representing non abundant ESTs obtained from 454 sequencing of normalized cDNA from tree peony buds during chilling treatment [19], and negative and positive control. It was generated following the manufacturer’s instructions. The raw data were) and the Project ID is 65,217.

Total RNA was amplified and labeled by Low RNA Input Linear Amplification kit (Cat#5184-3523, Agilent technologies, Santa Clara, CA, US), 5-(3-aminoallyl)-UTP (Cat#AM8436, Ambion, Austin, TX,US), and Cy3 NHS ester (Cat#PA13105,GE healthcare Biosciences, Pittsburgh, PA,US) following the manufacturer’s instructions. Labeled cRNA were purified by RNeasy mini kit (Cat#74106, QIAGEN, GmBH, Germany). Each Slide was hybridized with 1.65 µg Cy3-labeled cRNA using Gene Expression Hybridization Kit (Cat#5188–5242, Agilent technologies, Santa Clara, CA, US) in Hybridization Oven (Cat#G2545A, Agilent technologies, Santa Clara, CA, US), according to the manufacturer’s instructions. After 17 hours hybridization, slides were washed in staining dishes (Cat#121, Thermo Shandon, Waltham, MA, US) with Gene Expression Wash Buffer Kit(Cat#5188–5327, Agilent technologies, Santa Clara, CA, US), Stabilization and Drying Solution (Cat#5185–5979, Agilent technologies, Santa Clara, CA, US) following the manufacturer’s instructions. Slides were scanned by Agilent Microarray Scanner (Cat#G2565BA, Agilent technologies, Santa Clara, CA, US) and Feature Extraction software 10.7 (Agilent technologies, Santa Clara, CA, US) with default settings, with scan resolution = 5 µm, PMT 100%, 10%. Raw data were normalized by Quantile algorithm, Gene Spring Software 11.5.1 (Agilent technologies, Santa Clara, CA, US).

Differential expression was analyzed with a mixed model representation based on the normalized single channel intensities using the R software package LIMMA [20]. Significantly differentially expressed genes were identified (*P*<0.05) between any sample time points using ANOVA and Turkey’s HSD test. Redundancy of significantly differentially-expressed genes was eliminated before hierarchical clustering by calculating mean expression values for ESTs that aligned to the same contig. Cluster analysis of expression patterns for differentially expressed genes was done to identify likely endo-dormant and eco-dormant samples.

According to the analysis method of Mathiason [6], the expression data at 0 d of chilling treatment was not included in our study, which would reveal early response to chilling, not the accumulation of chilling treatment time. ANOVA identified significantly differentially expressed genes (*P*<0.05) with comparison between 6 d chilling treatment and other chilling time points. Pearson correlation hierarchical clustering was performed with MeV_4_0 software (http://mev.tm4.org) [21], to analyze gene expression patterns throughout the chilling requirement fulfillment periods.

To test the dormancy relevance of a subset genes involved in a specific biological process, we performed an unbiased gene ontology (GO) analysis and KEGG pathway analysis to make them be well-represented in an incomplete transcriptome sequencing effort as before described [19].The raw data have been deposited in NCBI’s Gene Expression Omnibus (GEO, http://www.ncbi.nlm.nih.gov/geo/) and are accessible through GEO Series number GSE40004.

### Real-time PCR

Candidate genes were chosen from redundant ESTs to ensure the availability of high quality sequence for primer design using Primer Premier 5.0 with default parameters for Real-time PCR (see File S1). Beta actin, which showed no significant change in expression throughout the chilling treatment, was used as control gene to normalize the RT-PCR results. RNA was extracted as described previously from flower buds that went through 0 d, 6 d, 12 d, 15 d, 18 d and 24 d of chilling treatments. First strand cDNA was synthesized from 2 µg of total RNA using PrimerScript™ RT reagent Kit (TaKaRa) according to the manufacturer’s instructions. The PCR reaction was performed in 25 µl, containing 12.5 µl 2×SYBR Green Master mix (TaKaRa), 300 nmol/L each primer and 2 µl 10-fold diluted cDNA template. The PCR reactions were run in Chromo 4 (BioRad, USA) using the following program: 95°C for 30 s and 45 cycles of 95°C 5 s, 57°C 30 s and 72°C 30 s. The reactions were run in triplicate, and the results for each gene were averaged. Heat-maps of the RT-PCR and microarray data were performed using Cluster and TreeView software (Stanford University, Stanford, CA, USA) as described by Eisen et al., based on log_2_ values [22].

## Results

### Evaluation of Dormancy Status of *Paoenia ostii* ‘Feng Dan Bai’

**Figure 1:**
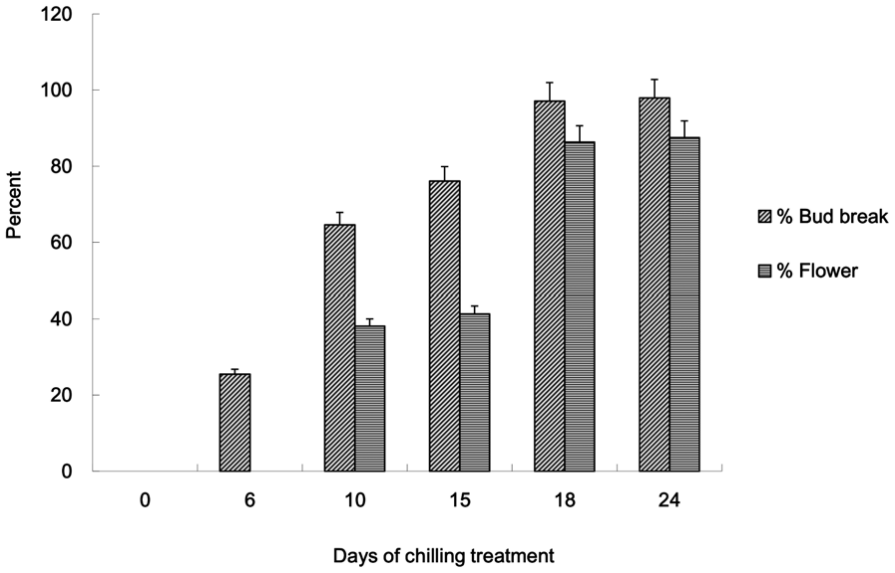
Morphologic changes of chilling treatment buds after incubated in green house (18–22°C, 8-h-light/16-h-dark cycle). Bud break was checked 30 days after moving to green house, and blossom was checked 60 days after that.

To determine the dormancy phases of buds, the fulfillment of the chilling requirement and the timing of transition from endo-dormancy to eco-dormancy in *P. ostii* ‘Feng Dan Bai’, we examined the percentages of budbreak and flower blossom (Figure 1). As the time of chilling treatment increased, the percentages of bud break and flower blossom increased with faster germination. After being transferred into greenhouse (18–22°C), the buds that received less than 6 d of chilling treatment faded away and never blossomed, whereas the buds that received about 12–15 d chilling treatment partially burst and then blossomed without normal leaves. After 18 d chilling treatment nearly 100 percentages of the buds sprouted and then blossomed. There was no difference between 18 d and 24 d of chilling treatment (*P*>0.05). The results indicated that the length of chilling treatment had a significant effect on the percentage of budbreak and blossom, and 18 d chilling treatment was required to completely break endo-dormancy in tree peony ‘Feng Dan Bai’. Therefore, flower buds receiving less than 18 d chilling treatment were regarded as endo-dormancy in physiological status, while those receiving more than 18 d chilling treatment were regarded as eco-dormancy.

### Gene Expression Profiles after Chilling Treatment

**Figure 2:**
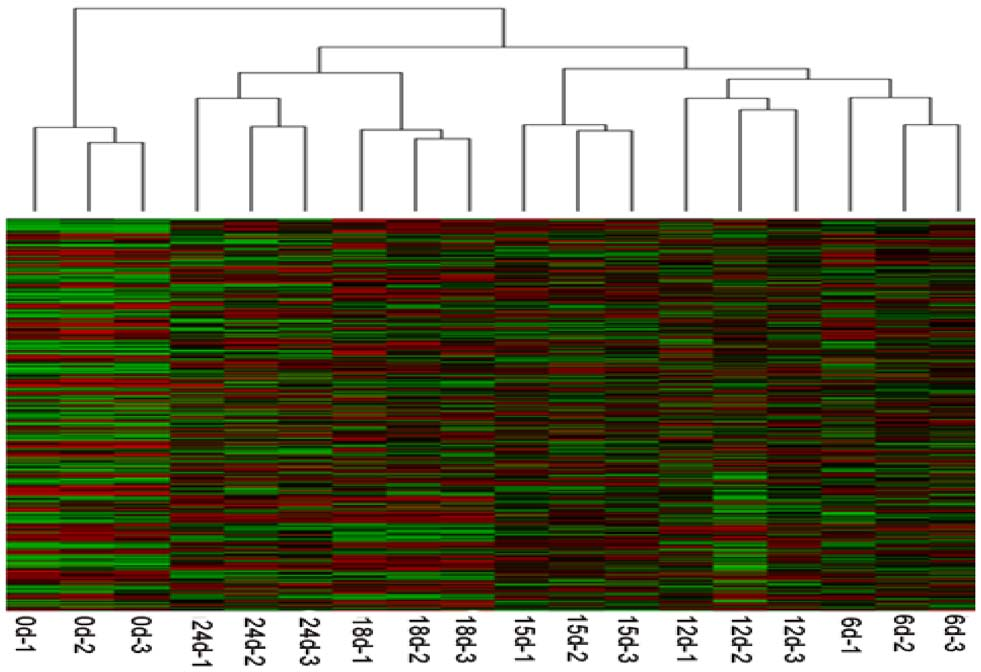
Cluster analysis of expression data from differentially-expressed genes. Relative gene expression levels shown with high expression are represented by red color and the low expression one are represented by green color.

Global mRNA profiling during chilling treatment was performed using customized Agilent tree peony Gene Chip. Significantly differentially-expressed genes were identified using ANOVA and Tukey's HSD (Honestly Significant Difference) test after comparison between any chilling treatments. From 14,951 different unigene probes which showed consistent hybridization, 3,606 genes were significantly (*P*<0.05) differentially expressed during chilling requirement fulfillment (See File S2, Table 1). The number of differentially expressed genes was relatively more by comparing 0 d and any chilling treatment time point, among which there were 1,367 and 1,002 differentially expressed genes between 0 d and 18 d, and 0 d and 24 d, respectively. Cluster analysis of these differentially expressed genes indicated that samples from different chilling treatments fell into two main groups (Figure 2). One group contained flowers buds collected from 0 d chilling treatment, the other cluster was divided into two subgroups, of which one subgroup contained flower buds from 18 d and 24 d chilling accumulation, and the other subgroup contained flower buds from 6 d, 12 d and 15 d chilling treatments. This result was also consistent with that of morphologic changes, which indicated flower bud receiving less than 18 d chilling treatment was in physiological status of endo-dormancy, while those receiving more than 18 d chilling treatment were in eco-dormancy physiological status.

**Table 1:** All pairwise comparisons of chilling treatments were computed using Tukey’ HSD Post Hoc test.

treatment of chilling days	24 d	18 d	6 d	0 d	12 d	15 d
24 d	3606	304	507	1002	485	255
18 d	3302	3606	292	1367	325	203
6 d	3099	3314	3606	429	60	135
0 d	2604	2239	3177	3606	738	955
12 d	3121	3281	3546	2868	3606	103
15 d	3351	3403	3471	2651	3503	3606

Entities found to be differentially expression in the top-right boxes, while entities found not to be differentially expressed represented in the lower-left boxes.

### Candidate Genes and Clustering

According to the analysis method of Mathiason [6], totally 3,174 genes were significantly differentially expressed by comparisons between 6 d chilling treatment and any other chilling treatments (See File S3).

**Figure 3:**
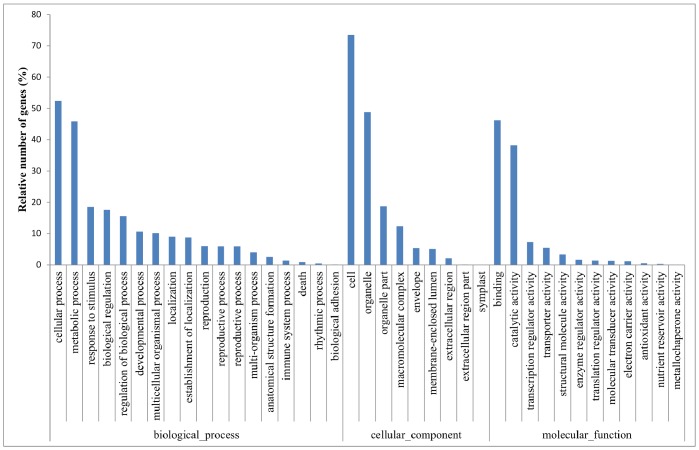
Functional category distribution of transcriptional changed genes in tree peony using Gene Ontology (GO). A comparison of 12 d, 15 d, 18 d and 24 d chilling treatment with 6 d chilling treatment yielded 3174 significantly differentially expressed features(*P*<0.05).

When comparing the expression data from 6 d, 12 d, 15 d, 18 d and 24 d of chilling treatments, the number of up-regulated (1,611) and down-regulated (1,563) of significantly differentially expressed microarray was almost the same (See File S3). Gene Ontology (GO) was used to gain a global overview of putative gene function. The above genes were assigned putative functions to Arabidopsis, and the results were shown in Figure 3. Across all chilling accumulation comparisons, totally 3,158 differentially expressed genes were similar to *Arabidopsis* genes. Most of the identified transcripts appeared to be genes involved in biological process, cellular component and molecular function. Biological processes involved in bud dormancy release were further analyzed using a division class approach described by Zhou et al [23], and cellular process (52.3%) and metabolic process (45.8%) were well-represented. The molecular function category was dominated by DNA binding (46.2%) and catalytic activity (38.1%), followed by transcription regulator activity (7.2%) and transporter activity (5.4%). There were 176 differentially expressed transcript factors and the majority of them was Myeloblastosis viral oncogene homolog (MYB), followed by Zinc finger protein, bHLH, HB, ARF and NAM, ATAF A and CUC (NAC) (Table 2). In addition, totally 16 differentially expressed genes could not be identified or classified. These genes might be novel tree peony genes or woody plant specific genes not yet annotated in the public databases.

**Table 2:** Categories and numbers of differentially expressed transcription factors during chilling duration(*P*<0.05).

Transcript factor	Number	Transcript factor	Number
MYB superfamily	18	DREB	2
Zinc finger protein	14	HMG	3
bHLH	14	MADS-box	1
HB	9	NAC	6
ARF	8	WRKY	5
bZIP	3	AP2	4
B3	3	F-box	3
GATA	4	others	79

**Figure 4:**
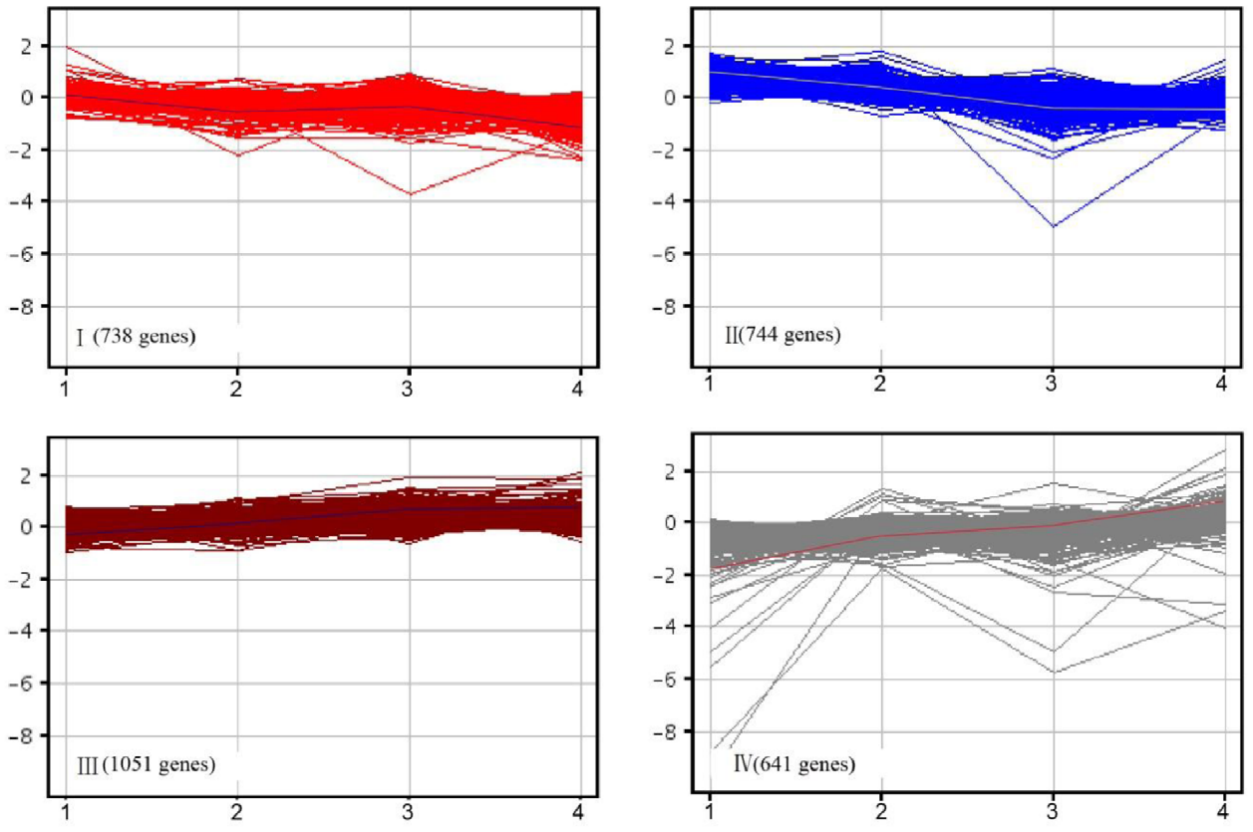
Clustering of significantly expressed genes during the chilling requirement fulfillment period. The x-axis represents days of chilling treatment, of which 1 is 6 d, 2 is the average of 12 d and 15 d, 3 is 18 d, and 4 is 24 d; y-axis shows fold-change (log scale).

The physiological status of flower bud after 12 d and 15 d chilling treatment was in the middle phase between endo-dormancy and dormancy release, the average value of 12 and 15 d chilling treatment was chosen as one time point. Hierarchical clustering of the expression ratios of four time points of accumulated chilling phases (6 d, average of 12 d and 15 d, 18 d and 24 d) versus the average value for each of the four phases indicated several expression patterns among differentially expressed genes. Cluster analysis was performed on averaged gene expression data from three biological replicates (See File S4). Four clusters of genes with coordinate expression were identified (Figure 4). Genes in cluster I and II were down-regulated during the early stage of chilling treatments. Totally 738 genes in cluster I exhibited rapid decrease from 6 d to 12 d chilling treatment, followed by minimal decrease until 18 d and then rapidly declined after 18 d chilling treatment. The expression level of four treatment time points were less than that at 6 d. There were 744 genes in cluster II which were dramatically down-regulated from 6 d to 18 d, followed by slightly decrease until 24 d.

Clusters III and IV contained genes that were up-regulated during the all stages of chilling treatments. Totally 1,051 genes were included in cluster III, which accounted for 33.1 percent of all differentially expressed genes. This group genes showed rapid increase from 6 d to18 d, following by steadily increase after 18 d. Totally 641 genes in cluster IV were dramatically up-regulated and showed rapid increase from 6 d to 24 d.

### Validation of Microarray Gene Expression Profiling

**Figure 5:**
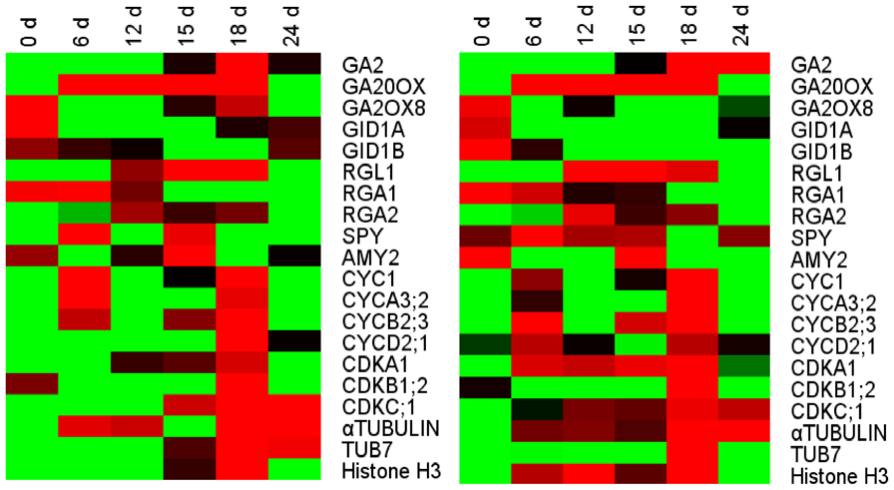
Heat map diagrams of gene expression detected by microarray analysis and RT-PCR analysis of interested genes. Green indicates down-regulation and red indicates up-regulation of the gene for particular treatment. Each column represents a treatment of 0 d, 6 d, 12 d, 15 d, 18 d and 24 d chilling. Averages of replicates from MA (left), averages of RTPCR (right).

Expression of differentially-expressed genes was confirmed by quantitative RT-PCR (Figure 5). Totally 10 candidate genes associated with GA signaling and response pathway (gibberellin 20-oxidase, gibberellin 2-beta-dioxygenase and GA2, etc.) and 10 candidate genes related to cell growth and development (CYCD2;1, CDKB1;2, histone H3 and TUB7, etc.) were analyzed. One key enzyme of GA biosynthesis in the GA-response pathway [24], a putative GA 20-oxidases gene (*GA20ox*), was significantly up-regulated at 6 d chilling treatment and decreased after endo-dormancy release. A *GA2ox-*like gene was dramatically down-regulated after 6 d chilling exposure. A putative ent-kaurene synthase gene (*GA2*) increased after 18 d chilling duration. In addition, the putative CYC members (CYCA, CYCB and CYCD) and CDK members (CDKA, CDKB and CDKC) were peak just at the time point of 18 d (end of endo-dormancy) chilling treatment. These results suggested that the general trends in expression obtained by microarray analysis were similar to that tested by RT-qPCR.

### Genes Associated with GA Response and Signaling Pathway during Dormancy Release

GA had been known to modulate development throughout the plant life cycle, and exogenous GA could effectively promote endo-dormancy release and accelerate bud sprouting in tree peony [16]. Based on the results of Gene Ontology (GO) analysis, there were totally 51 probes associated with GA response (GO: 0071370) and GA signaling pathway (GO: 0009740) (See File S5), among which 22 genes were differently expressed. There were 12 differently expressed genes involved in signaling pathway mediated by gibberellic acid including *GA2*, *GA20ox*, *GA2ox*, *RGA1* and *SPINDLY* (*SPY*).

## Discussion

Tree peony became endo-dormant when photoperiod and temperature decreased at the end of the growing season and sufficient chilling was the main factor for dormancy release and growth recovery in the spring. Although chilling days had been reported for tree peony (*P. suffruticosa*) cultivars ‘Lu he hong’ [17], [18], no such information was available for *P. ostii* ‘Feng Dan Bai’. The results of this study indicated that 18 d chilling were sufficient to break bud dormancy in ‘Feng Dan Bai’. When exposed to less than 18 d chilling treatment, the percentages of bud break and flower blossom was low. Cluster analysis of these differentially expressed genes also tested the conclusion of morphologic changes. Examination of the expression data from 6 d, 12 d, 15 d, 18 d and 24 d of chilling treatments revealed that the number of up-regulated and down-regulated of significantly differentially expressed microarray was almost the same. This trend was different from that observed in *Arabidopsis* [25], sunflower [26], *Vitis riparia* [6] and leafy spurge [27]. The difference might be related to the biological and ecological characteristics of different species.

### GA Synthesis and Signaling in Dormancy Release

Zheng et al found that exogenous GA could effectively promote endo-dormancy release and accelerate bud sprouting in tree peony [16]. Totally 22 genes related to GA signaling pathway and GA response were differently expressed during chilling treatment (See File S5). The result indicated the involvement of GA during dormancy release. A putative GA 20-oxidases gene (*GA20ox*), which encodes a key protein for bioactive GAs biosynthesis [24], [28], was significantly up-regulated at 6 d chilling treatment and decreased after endo-dormancy release, and a putative *GA2ox* gene, whose protein can inactivate most active GAs [29], was dramatically down-regulated after 6 d chilling exposure. The more available transcripts of *GA20ox* through repressing *GA2ox,* might subsequently elevate the contents of GA in dormancy buds as reported by Zheng et al [16]. Furthermore, a putative ent-kaurene synthase gene (*GA2*, cluster III) which functions in the first step of GA biosynthesis, increased after 18 d chilling enduring. These results were also tested by RT-qPCR. GA biosynthesis is subject to feedback regulation by the GA-response pathway, which might be the reason of the transcriptional decrease of *GA20ox* near bud break. DELLA proteins are negative regulators of GA-induced growth. Several putative *DELLA*s genes were differentially expressed during chilling treatment, among which *RGA1* (cluster I) were significantly down-regulated. In Arabidopsis, five DELLA proteins (GAI, RGA, RGL1, RGL2, and RGL3) were identified with some overlapping and unique functions [30]. It was hypothesized that DELLA played important roles in maintenance of well-defined phases of dormancy in leafy spurge [9], which was consistence with our results.

**Figure 6:**
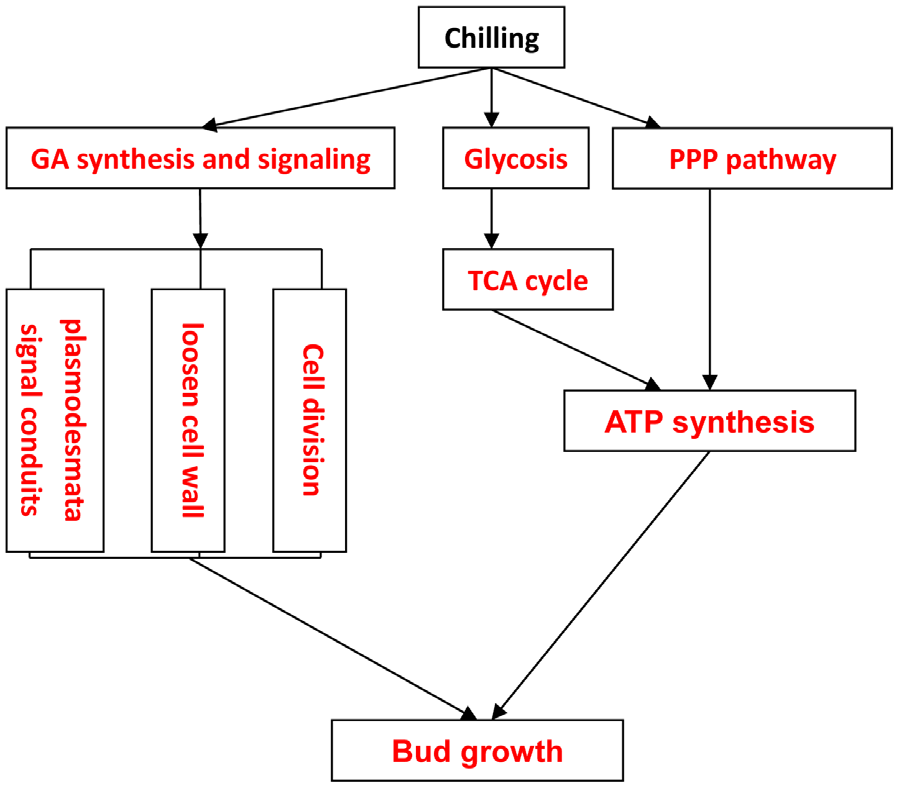
Hypothesis of reprograming during chilling induced dormancy release-model of tree peony. Red character represents up-regulation.

GA-induced α-amylase gene expression in aleurone cells has been a useful model system to study GA response pathway [24]. The chilling-induced putative α-amylase transcript (*AMY2*, cluster IV) might be related to GA signaling. Additionally, two putative β-1, 3-glucanase encoded genes (*GH9A1* in cluster IV, and *AT3G23600*, cluster III) and one chitinase encoded gene (*POM1*, cluster III) were up-regulated close to dormancy release. β-1,3-glucanase and chitinase, GA-dependent enzymes, could loosen cell wall by hydrolyzing cell wall components and facilitate cell growth and elongation [31]. Rinne et al reported that chilling could recruit GA-inducible β-1, 3-glucanase to reopen signal pathway and release dormancy in *populus* [32]. These results implicated that the activation of GA biosynthesis and signaling pathway by artificial chilling could subsequently induce cell growth and dormancy release [33]. Therefore, we hypothesized that GA played a key role in chilling induced dormancy release in tree peony (Figure 6).

### Antioxidant Activity

Previous studies indicated that oxidative stress accompanied with the response to artificial and chilling dormancy breaking stimuli in deciduous fruit tree buds [4], [6], [8], [34]–[36]. Moreover, exogenous application of H_2_O_2_ promoted budbreak, suggesting the involvement of H_2_O_2_ in dormancy release of grapevines [36]. Our data showed the transcript of catalase (*CAT1*, cluster I) was reduced during chilling treatment, and members of the antioxidative machinery such as glutathione reductase (*GR1*, cluster III), glutathione peroxidase (GPX2, cluster III), ascorbate peroxidase (*APX4*, cluster IV), glutathione S-transferase (*DHAR2* and *DHAR3*, cluster III) and peroxidase (AT3G17070, cluster IV) were induced and grouped to cluster III and IV. Up-regulated expression of *APX*, *GR* and *GST* indicated that the ascorbate-glutathione cycle was probably induced to cope with the increased oxidative stress in the bud during chilling treatment. The results were consistent with our previous report of the related enzymes activities in the same process [15], and similar to other reports in grape, apple and kiwifruit etc. [8], [35], [37]. However, exogenous application of H_2_O_2_ or paraquat could not promote bud break, but exogenous ascorbic acid could shorten the bud sprouting time (Gai et al, unpublished data). Therefore, increase of H_2_O_2_ content might not be the primary signal to bud dormancy break and subsequent bud sprouting in tree peony.

### Carbohydrate and Energy Metabolism

Carbohydrates are the main source of energy for metabolic changes that occur during dormant sprouting and blossoming in trees [38]. Starch serves as an important carbohydrate reserve from the final leaf drop to several weeks after budbreak [38]. During dormancy, the bud exhibited decrease absorption potential and increased soluble sugar concentration through starch hydrolysis [39]. A putative α-amylase (similarity to *Arabidopsis AMY2*, cluster IV) was significantly induced during the chilling requirement period and reached peak at 18 d chilling, and a putative β-amylase 1 (*BAM1* cluster III) was also up-regulated after 18 d chilling treatment. These results agreed with the downward tendency of starch concentrations in dormant buds [14]. The majority of genes related to Glycolysis, Citrate cycle (TCA cycle) and Pentose phosphate pathway (PPP) were clustered into cluster III and IV, which represented up-regulated genes during chilling treatment. Three key glycolytic enzymes encoded genes hexokinase (*HXK3*, cluster III), 6-phosphofructokinase (*PFK3*, cluster III) and pyruvate kinase (*AT2G36580*, cluster III, and *PKP*, cluster III) were up-regulated after 15 d chilling treatment, which indicated activation of glycolysis pathway at the later stage of endo-dormancy. These results were not consistent with the response of grape and other dormant plants to chilling and artificial treatments [7], [35], [40]. Pyruvate dehydrogenase component E1 (putative *MAB1* and *PDH-E1* encoding α and β subunit respectively), which are responsible for entry of pyruvate into the TCA cycle, were induced near dormancy release [7]. But several other enzymes that are involved in subsequent steps, such as isocitrate dehydrogenase, 2-oxoglutarate dehydrogenase, succinate dehydrogenase, malate and NAD-malate dehydrogenase were slightly up-regulated close to budbreak. These data indicated that TCA pathway was induced by chilling treatment in tree peony, coinciding with the enzymes activities during chilling induced dormancy release in *Dioscorea esculenta* and *Curcuma longa* [41]. Pentose phosphate pathway was also activated during chilling treatment, the increase of glucose-6-phosphate dehydrogenase (*G6PDH*, cluster IV) and 6-phosphogluconolactonase 1 (puatative *AT3G02360*, cluster III) were evident during the late chilling stages. It was reported that PPP pathway activation was associated with dormancy release both in perennial bud and seed [41], [35]. Bud meristems require high import of sugars from the underlying tissue, which is essential to sustain bud growth at the time of dormancy release [37]. Besides the activation of carbohydrate metabolism, the transcriptional level of a putative sucrose transporter (*SUT4*, cluster III) also increased at the 18 d chilling time point. Changes of carbohydrate metabolism above could meet the substance and energy requirements for bud break and subsequent sprouting.

Respiratory stress was regarded as one of effective inducers of grape bud break, such as significant induction of pyruvate decarboxylase (*PDC*) and alcohol dehydrogenase (*ADH*) genes, and reduction of oxidative phosphorylation pathway induced by chilling and artificial stimuli [4], [34], [35], [42]. In addition, the similar results were also found in the transcriptome of raspberry and kiwifruit [8], [37]. On the contrary, putative alcohol dehydrogenase (*ADH*, cluster I) gene expression was significantly reduced after chilling treatment in tree peony. Moreover, the increase of succinate dehydrogenase (cluster III) expression indicated that high mitochondrion activity was induced near the end of dormancy. The discrepancy did not support the hypothesis that similar mechanisms of dormancy release were employed by buds from different species in response to various stimuli [42]. Therefore, differences might exist in mechanism of endo-dormancy between tree peony and the fruit trees, for example the response to exogenous stimulus (for example HC and GA_3_) was diverse between tree peony and other fruit trees (apple, grape, kiwifruit and raspberry, etc.) [4], [8], [34], [35], [37], [42].

Besides induction of carbohydrate metabolism, oxidative phosphorylation was also activated close to budbreak. Our data showed that one or more members encoding subunits of oxidative phosphorylation pathway complexes I to V, such as NADH dehydrogenase, succinate dehydrogenase, cytochrome c oxidase and ATP synthase, were induced following chilling treatment in our data. The buds that entered dormancy contained very low level of ATP, and the ratio of ATP/ADP is often used to indicate the status of dormancy and the growing capacity of buds/seeds [43], [44]. In previous study, we demonstrated that increase of *PsMPT* expression could promote the output of ATP content and dormancy release in tree peony [18]. Here, significant activation of carbohydrate metabolism and oxidative phosphorylation during dormancy release are consistent with the phenomena of ATP increase.

### Cell Division and Growth

Following the breaking of dormancy, vegetative growth was often combined with the increasing of cell division. Changes in cell cycle-specific gene expression occurred during release of axillary buds of pea [45] and potato [46], and adventitious buds of leafy spurge [47], such as up-regulation of D-type cyclins (CYCD) acting at the G1-S-phase transition resulted in dormancy breaking [48]. During the transitions from endo-dormancy to eco-dormancy in tree peony, 114 differentially expressed genes were identified to involve in cell cycle and growth, and nearly half (56/114) of them were up-regulated during chilling treatment. A putative CYCD (cluster III) member, which is regulated by GA and other signal substrate [49], was strongly up-regulated after 18d chilling exposure. Moreover, the transcripts of putative CYCA, CYCB and CYKD, key regulator of transition from G2 to M phase in cell division, were peak just at the time point of 18 d (end of endo-dormancy) chilling treatment. These results were similar to recent reports in leafy spurge [5], [50]. The results might indicate that cell division was gradually reinitiated and accelerated at the end of endo-dormancy.

Eukaryotic DNA is packed into chromatin, the basic unit of which is nucleosome, consisting of DNA wound around histone protein complexes. Total 9 Histone were differently expressed, of which the members of Histone (HisH2a, HisH2b and HisH4) were inhibited during chilling treatment. However, HisH3 was up-regulated before 18 d and down-regulated after 18 d chilling treatment. Combining with physiological status, the timing of 18 d chilling treatment was defined as late time point in endo-dormancy or early time point in eco-dormancy [51]. Anderson et al also found that HisH3 transcript levels were up-regulated during para-dormancy and most of endo-dormancy, but were down-regulated during the later phase of endo-dormancy and through eco-dormancy [50]. The results of Histone expression indicated that inhibition of growth during endo-dormancy was not a consequence of reduced or increased transcript availability.

*HUB1*(cluster I) encoding a C3HC4 RING finger domain ubiquitin E3 ligase was up-regulated before 18 d treatment, but down-regulated after 18 d chilling treatment. HUB1 is required for HisH2b monoubiquitination [52], and monoubiquitination is a key modification associated with transcriptional active chromatin. In *Arabidopsis*, *hub1* mutant had reduced seed dormancy and altered expression levels for several dormancy-related genes [53]. Induction of HUB1 might accelerate the release of endo-dormancy by HisH2b monoubiquitination in tree peony.

### Plant-growth Regulators, Ethylene, Auxin and ABA are also Involved in Dormancy Modulation

Plant hormones such as ABA (abscisic acid), auxin and GA play a significant role in endo-dormancy induction and release. Recent study showed that ethylene played a role in terminal bud development and endo-dormancy induction in birch [54]. In grape, Ophir et al reported the new relation between ethylene metabolism and cell enlargement during bud dormancy release [7]. The integral membrane protein ETHYLENE-INSENSITIVE2 (EIN2) was a central regulator of all ethylene responses, and expression of EIN2 was sufficient to constitutively activate ethylene responses, and induced downstream ethylene responsive factors (ERF) including dehydration responsive element binding-protein (DREB), cold-binding factor (CBF) and AP-binding elements [55]. Ethylene induction might initiate a physiological chain of events that lead to growth cessation and dormancy. *EIN2* (cluster II) was steadily up-regulated early chilling requirement fulfillment period, and quickly down-regulated from 12 d to 28 d chilling treatment. Additionally, 9 putative ERF genes were differentially expressed during chilling treatment, indicating ethylene might be involved in the regulation of dormancy release in tree peony (See File S4). In *Arabidopsis*, the ethylene-insensitive mutants *etr1* and *ein2* had a late blossom phenotype [56], [57], suggesting that ethylene might facilitate the transition to blossom, a process that requires reprogramming of the vegetative SAM. In our study, the *EIN2* was induced early in chilling treatment, which might indicated that meristem reprogramming also occurred during growth cessation and the development of bud dormancy.

It had been demonstrated that auxin metabolism and signaling were central regulators of para-dormancy [5], [9], [51], [58]. The role of auxin in endo-dormancy is still unknown. Auxin transport was reduced during the transition to endo-dormancy in lead spurge [50], implying the function of auxin in dormancy regulation. In our study, 8 genes with blast hits to auxin response factors (ARF) and auxin transporter showed differential expression during chilling treatment. These data suggested that auxin involved in the regulation of dormancy in tree peony.

ABA had been implicated to function in the induction of endo-dormancy [5], and its level and signaling were then repressed by prolonged chilling [5], [9], [16]. Putative *Arabidopsis* homologues involved in ABA signaling and response (*PYL11*, *HVA22a*) were also differentially expressed in our microarray data. Ethylene could induce ABA biosynthesis [59], while ABA antagonized GA, and GA accumulation was often associated with plant cell elongation and cell division [60]. Thus, crosstalk between GA, ABA and ethylene might regulate endo-dormancy in tree peony.

### Genes Responding to Abiotic Stimulus

Totally 233 genes responding to abiotic stimulus were differentially-expressed, of which two encoded calmodulin-binding transcription activator. One of the calmodulin-binding transcription activator was homologous to WRKY and the other similar to CDPK-related kinase, and both of them were down-regulated in early chilling treatment. Calmodulin-binding proteins exist in many different forms [61], and have been demonstrated to control calcium signaling in woody perennial species such as blackcurrant [10]. In grape, induction of CDPK was identified after artificial induction of dormancy release by hydrogen cyanamide (HC) [62]. The data suggested that calcium signaling might be involved in the mechanism of bud dormancy release.

Genes encoding CRT-binding factor (*CBF*, cluster IV) and dehydration-responsive element-binding protein (*DREB*, cluster IV) were up-regulated throughout chilling treatment periods. The CBF/DREB1 transcription factors control an important pathway for increasing abiotic stress tolerance, such as freezing and drought tolerance in plant. Constitutive expression of *CBF* in transgenic *Arabidopsis* plants resulted in an increase in freezing and drought tolerance [63], [64]. In *Vitis*, endogenous CBF was enhanced upon exposure to low temperature. It was likely that up-regulated expression of *CBF* and *DREB* with *EIN2* helped to increase freezing tolerance in tree peony after chilling treatment.

### Conclusions

Adequate artificial chilling alone is an effective way to break dormancy and promote subsequent growth and flower blossom in tree peony. The transcriptome profiles described in this paper provided clear relationships between chilling treatment and gene expression. Genes involved in GA biosynthesis and signaling, carbohydrate metabolism, energy metabolism, transporter, cell division and growth were well represented in the differentially expressed genes. In tree peony forcing culture practice, exogenous application of GA can quickly break bud dormancy, and greatly shorten the germination timespan comparing to chilling induced dormancy break. Yamauchi et al (2004) reported that low temperature could activate gibberellin biosynthesis and response pathways [33]. Our previous report showed accumulation of GA when dormant tree peony was chilling-treated [16]. Our microarray data also demonstrated that key genes related to GA biosynthesis and signaling pathway were quickly induced by artificial chilling treatment. Subsequently, GA-dependent enzymes, β-1,3-glucanase, chitinase and α-amylase were activated to recruit plasmodesmata signal, loosen cell wall, and hydrolyze starch. Carbohydrate metabolism, such as Glycolysis, Citrate cycle (TCA cycle) and Pentose phosphate pathway (PPP) were induced at the end of endo-dormancy. Similar trend was observed in oxidative phosphorylation, indicating enhanced ATP production at dormancy-breaking stage. ATP level is essential for dormancy release and sequential bud break [18]. The activation of carbohydrate metabolism and oxidative phosphorylation can promote ATP accumulation, and prepare for the following bud sprouting.

## Supporting Information

File S1 Primer sequences for Real-time PCR. Real-time PCR primers were designed using Premier Primer 5.0 software. Information on the name of the sequences containing homologue, primer sequences, Tm and expected length of PCR product was included.
(XLS)

File S2 Significantly differentially-expressed genes were identified using Tukey's HSD (Honestly Significant Difference) test (*P*<0.05). Information of probename, Genbank accession number, description, E-value, objected, normalized expression value using Tukey’s HSD test was included.
(XLS)

File S3 Significantly differentially-expressed genes were identified using the analysis method of Mathiason. Total 3,174 genes were significantly differently expressed by comparisons between 6 d chilling and any other chilling, and the expression data at 0d of chilling was not included. Information of probename, Genbank accession number, description, E-value, objected, expression values was included.
(XLS)

File S4 Cluster analysis based on averaged gene expression data from three biological replicates. The information of probeId, Genbank accession number, description, E-value, cluster, symbol, subcategory, category and the relative expression ratios of four time points of accumulated chilling phases (6 d, average of 12 d and 15 d, 18 d and 24 d) versus the average value for each of the four phases was included.
(XLS)

File S5 List of probes and differentially-expressed probes related to GA response and signaling pathway. The information of probeID, GeneID, GO subcategory, differentially expressed or not and symbol was included.
(XLS)
